# Allosteric DNA nanoswitches for controlled release of a molecular cargo triggered by biological inputs†
†Electronic supplementary information (ESI) available: Supporting information. See DOI: 10.1039/c6sc03404g



**DOI:** 10.1039/c6sc03404g

**Published:** 2016-11-03

**Authors:** Marianna Rossetti, Simona Ranallo, Andrea Idili, Giuseppe Palleschi, Alessandro Porchetta, Francesco Ricci

**Affiliations:** a Chemistry Department , University of Rome Tor Vergata , Via della Ricerca Scientifica , Rome 00133 , Italy . Email: francesco.ricci@uniroma2.it ; Email: alessandro.porchetta@uniroma2.it

## Abstract

Here we demonstrate the rational design of a new class of DNA-based nanoswitches which are allosterically regulated by specific biological targets, antibodies and transcription factors, and are able to load and release a molecular cargo (*i.e.* doxorubicin) in a controlled fashion. In our first model system we rationally designed a stem-loop DNA-nanoswitch that adopts two mutually exclusive conformations: a “Load” conformation containing a doxorubicin-intercalating domain and a “Release” conformation containing a duplex portion recognized by a specific transcription-factor (here Tata Binding Protein). The binding of the transcription factor pushes this conformational equilibrium towards the “Release” state thus leading to doxorubicin release from the nanoswitch. In our second model system we designed a similar stem-loop DNA-nanoswitch for which conformational change and subsequent doxorubicin release can be triggered by a specific antibody. Our approach augments the current tool kit of smart drug release mechanisms regulated by different biological inputs.

## Abstract 2

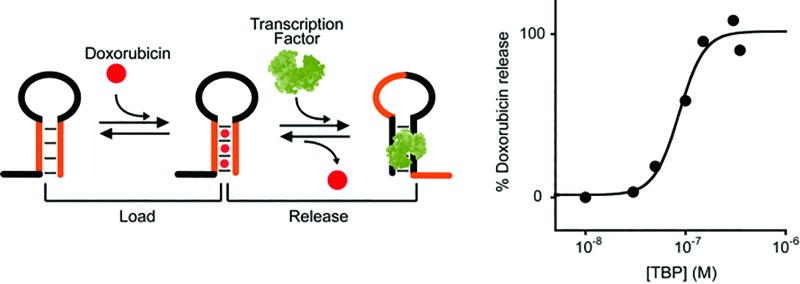
A rationally designed new class of DNA-based nanoswitches allosterically regulated by specific biological targets, antibodies and transcription factors, can load and release a molecular cargo in a controlled fashion.

## Introduction

Over millions of years of evolution, Nature has optimized an incredible and complex network of interactions and signalling pathways for controlling a wide range of cellular activities.^1^ In response to different environmental stimuli, for example, proteins can perform a huge number of functions ranging from the catalysis of metabolic reactions^2^ to molecular transport^3^ and gene expression.^4^ Despite the large variety of such functions and the biological inputs employed, Nature relies on a limited number of methods to control, turn-on or shut-down the activity of such functional biomolecules. One of the most used of these methods is allostery, a mechanism through which the binding of an effector at one site of the functional receptor causes a conformational change that affects (activates or inhibits) its activity.^5^

Allosteric control has been demonstrated in a large number of biomolecular systems and underlies so many cellular and protein functions that it is believed that nearly all proteins display some level of allosteric behaviour.^6^ For example, key proteins such as myosin,^7^ G protein-coupled receptors,^8^ and phosphofructokinase^9^ employ allostery to control their functions. Similarly, riboswitches employ allosteric rearrangement of a mRNA structure mediated by ligand binding to modulate gene expression.^10^ Finally, allostery is also strongly involved in processes that allow the transport of molecular cargoes across the cell.^11^ The best example of this kind is the allosteric control of hemoglobin by 2,3-bisphosphoglycerate (BPG). This small molecule binds to hemoglobin and decreases the protein's affinity for oxygen thus enhancing oxygen transport efficiency.^12^

Because of the versatility of the allosteric mechanism in controlling different biochemical functions, recreation of *in vitro* allosterically-regulated receptors is one of the main goals in the field of synthetic biology and biotechnology for the development of “smart” biomaterials,^13^ novel theranostic tools,^14^ and drug-release devices with controlled features.^15^ Our group, following the seminal work of Famulok, Breaker and others,^16^ has recently demonstrated the usefulness of re-creating allostery *in vitro* to rationally control the dynamic range of DNA-based switches for biosensing purposes.^17^ Moreover, recent studies have demonstrated the crucial role of allosterically controlled RNA in the cell in both silencing and activating gene expression.^18^

**Fig. 1:**
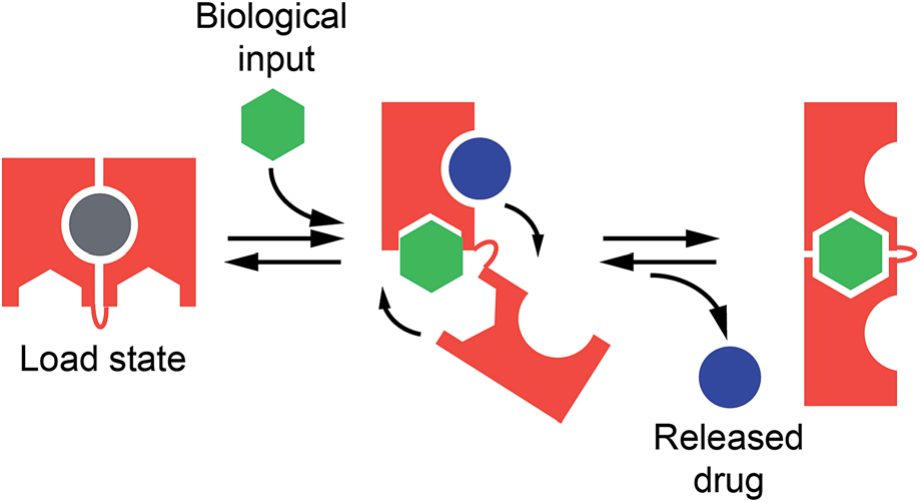
Nature often employs allostery to regulate the affinity of biomolecules and, through this, many biological functions including target recognition and molecular transport. Inspired by this mechanism, here we propose to rationally design allosterically regulated DNA-nanoswitches (red) that can load a molecular cargo (circle) and release it upon binding to a macromolecular allosteric effector (green hexagon).

Despite the above efforts, however, the use of allosteric control to develop synthetic machines that are able to load and release a molecular cargo in a way similar to that used by naturally-occurring molecular transporters has seen very little application.^19^ Prompted by the above arguments, here we demonstrate a new class of allosterically regulated DNA-based nanoswitches that are able to release, in a controlled fashion, a molecular cargo in response to the binding of a biomolecular effector (Fig. 1). To do this we have selected two classes of relevant disease markers (*i.e.* antibodies and nucleic-acid-binding proteins). As a proof of principle of this strategy we employed, as the cargo molecule, doxorubicin, which is an anthracycline that interacts with B-DNA structures through the intercalation of the anthraquinone moiety at the GC portion of the oligonucleotide sequence,^20^ and is one of the most effective and broad spectrum anticancer drugs used in the treatment of different solid tumors.^21^

## Results and discussion

**Fig. 2:**
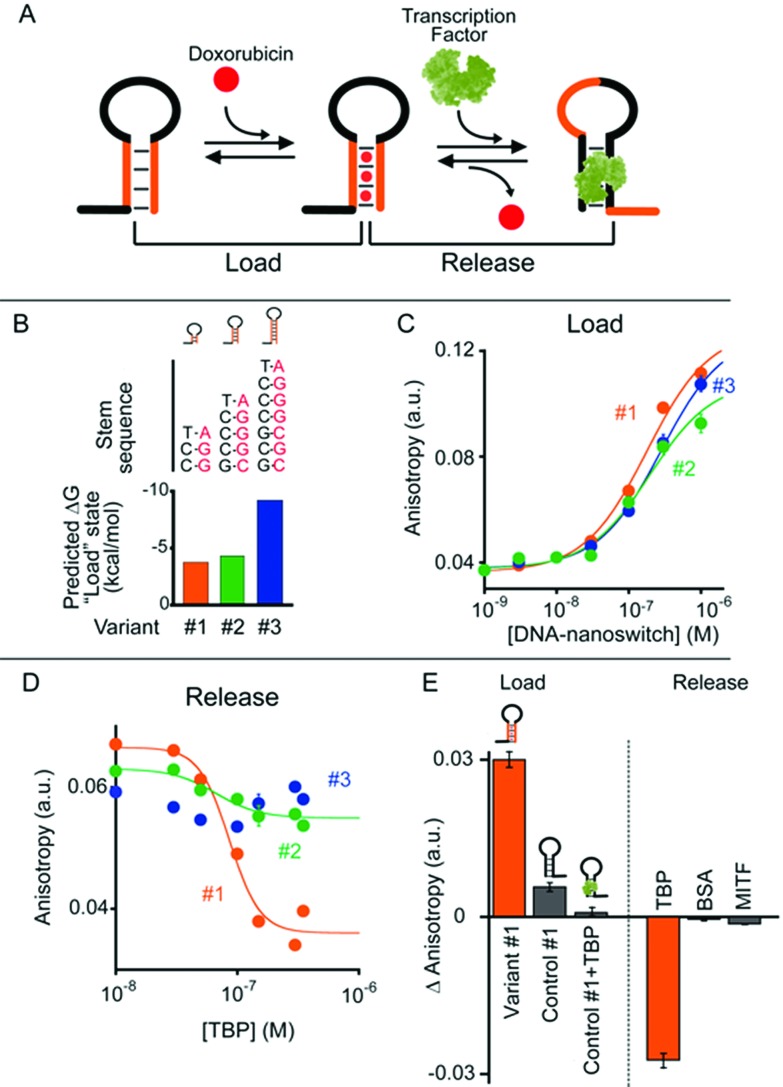
(A) TATA-binding protein (TBP)-regulated DNA-nanoswitches coexist in two mutually exclusive stem-loop conformations (*i.e.* “Load” and “Release”). The binding of TBP to the latter conformation pushes this conformational equilibrium towards the “Release” state, thus triggering the release of the drug (*i.e.* doxorubicin) intercalated in the “Load” state. (B) We engineered three variants of DNA-nanoswitches with increasing stabilities of the “Load” state (indicated are the free energies predicted using mfold). (C) All three variants show a similar affinity for doxorubicin. (D) Drug release efficiency upon TBP addition for the three DNA-nanoswitch variants. (E) Variant #1 shows good loading efficiency while a control DNA-nanoswitch which contains the TBP recognition element but lacks the GC-rich stem portion (control #1) shows a much lower loading efficiency towards doxorubicin intercalation, especially when previously bound to TBP. Variant #1 also shows high specificity against other, non-specific proteins (at 350 nM). Doxorubicin load/release experiments have been performed by measuring doxorubicin fluorescence anisotropy signals. The excitation and emission wavelengths were fixed at 480 nm (±7 nm) and 592 (±10) nm, respectively. For loading experiments, increasing concentrations of the relevant DNA-nanoswitch were added to a solution of 100 nM of doxorubicin. For releasing experiments, increasing concentrations of TBP were added to an equimolar solution of doxorubicin and the DNA-nanoswitch (100 nM). (E) Loading results were also obtained with an equimolar solution of doxorubicin and the DNA-nanoswitch or control (100 nM). All experiments were conducted at pH 7.0 in 50 mM sodium phosphate, 150 mM NaCl and 10 mM MgCl_2_ in a 100 μL cuvette at 25 °C. For further information about the experimental set up see the Experimental section.

In our first model system we designed a stem-loop DNA-nanoswitch that can adopt two mutually exclusive conformations: (i) a “Load” conformation containing a doxorubicin-intercalating stem domain (orange stem, Fig. 2A) and (ii) a “Release” conformation containing a duplex stem (black stem, Fig. 2A, right) that is recognized by a specific transcription-factor (here Tata Binding Protein, TBP). The binding of the transcription factor pushes this conformational equilibrium towards the latter (“Release”) state thus leading to the release of the drug intercalated in the “Load” state (Fig. 2A). Because the intercalation of doxorubicin in DNA can be easily followed through its fluorescence anisotropy signal change (increase) this provides a means to measure doxorubicin load/release from our DNA-nanoswitch.^22^

We first designed three variants of TBP-regulated DNA-nanoswitches, each having the same consensus binding sequence for TBP (black stem, Fig. 2A, right) but differing in the stability of their “Load” state. To do so, we increased the GC pair content of the stem (orange stem, Fig. 2A) and obtained variants with estimated free energies of the “Load” state (Δ*G*_load_state_) ranging from –9.2 to –3.7 kcal mol^–1^ (values predicted using mfold prediction software) (Fig. 2B).^23^ Because the energy of the “Release” state is effectively identical in all three variants (Δ*G*_release_state_ = –4.6 kcal mol^–1^), this approach provides a means of tuning the switching equilibrium constant between the load/release states and thus the concentration range of TBP over which the DNA-nanoswitch mediated release of doxorubicin will occur. By titrating doxorubicin with increasing concentrations of the three variants we observe a similar trend in the concentration-dependent increase of the doxorubicin anisotropy signal which suggests a similar doxorubicin intercalation efficiency for all three variants (Fig. 2C).

The release of doxorubicin from the DNA-nanoswitch is regulated by TBP. To demonstrate this, we added to an equimolar solution of doxorubicin and the DNA-nanoswitch increasing concentrations of TBP, and followed the doxorubicin fluorescence anisotropy signal decrease, which ultimately provides a means of measuring doxorubicin release from the DNA-nanoswitch. For the variant with the lowest stability of the “Load” state (*i.e.* variant #1, Fig. 2B) we observe a monotonic doxorubicin release with increasing TBP concentrations and an EC_50_ (the concentration of TBP at which 50% of loaded doxorubicin is released) of 80 ± 10 nM (Fig. 2D). Of note, at saturating concentrations of TBP (*i.e.* 350 nM) we observe a complete release of the loaded doxorubicin from the DNA-nanoswitch. By using the doxorubicin-nanoswitch titration curve discussed above (Fig. 2C), we can thus estimate the total maximum amount of doxorubicin released from the DNA-nanoswitch upon TBP binding (*i.e.* 3.2 pmol in a 100 μL volume). As expected, variants with a higher stability of the “Load” state show a different degree of doxorubicin release. Variant #2, for example, shows only partial (*i.e.* 32 ± 4%) doxorubicin release at the saturating concentration of TBP (*i.e.* 350 nM). Furthermore, because the high stability of the “Load” state for variant #3 does not allow the conformational change even at saturating concentrations of TBP, we did not observe any doxorubicin release under these conditions using this variant. A control experiment showing the absence of any specific interaction between free doxorubicin and TBP further suggests that the observed decrease in doxorubicin anisotropy upon TBP addition is due to the actual release of doxorubicin following the TBP-induced conformational change of the nanoswitch (Fig. S1†).

To further support these results, we have employed a control system (control #1) that only contains the TBP consensus sequence and is thus forced into the “Release” state. Because this system does not have any GC base pairs (which are known to favour doxorubicin intercalation) the observed affinity towards doxorubicin is much lower than that observed with the “Load” state (Fig. 2E, control #1). Moreover, if this control system is previously incubated with TBP, no significant intercalation of doxorubicin is observed (Fig. 2E, control #1 + TBP). Finally, a control experiment has been performed using a DNA-nanoswitch (*i.e.* control #2) which has the same doxorubicin-intercalating stem but lacks the TBP recognition element in the sequence. As expected, such a control did not show any doxorubicin release upon TBP addition (Fig. S2†). Specificity tests performed on the best performing TBP-regulated DNA-nanoswitch (variant #1) did not show any significant doxorubicin release when challenged with saturating concentrations of other non-specific proteins (Fig. 2E). Moreover, a similar load/release efficiency has been observed for the same variant over a wide temperature range (from 25 to 40 °C) (Fig. S3 and S4†).

**Fig. 3:**
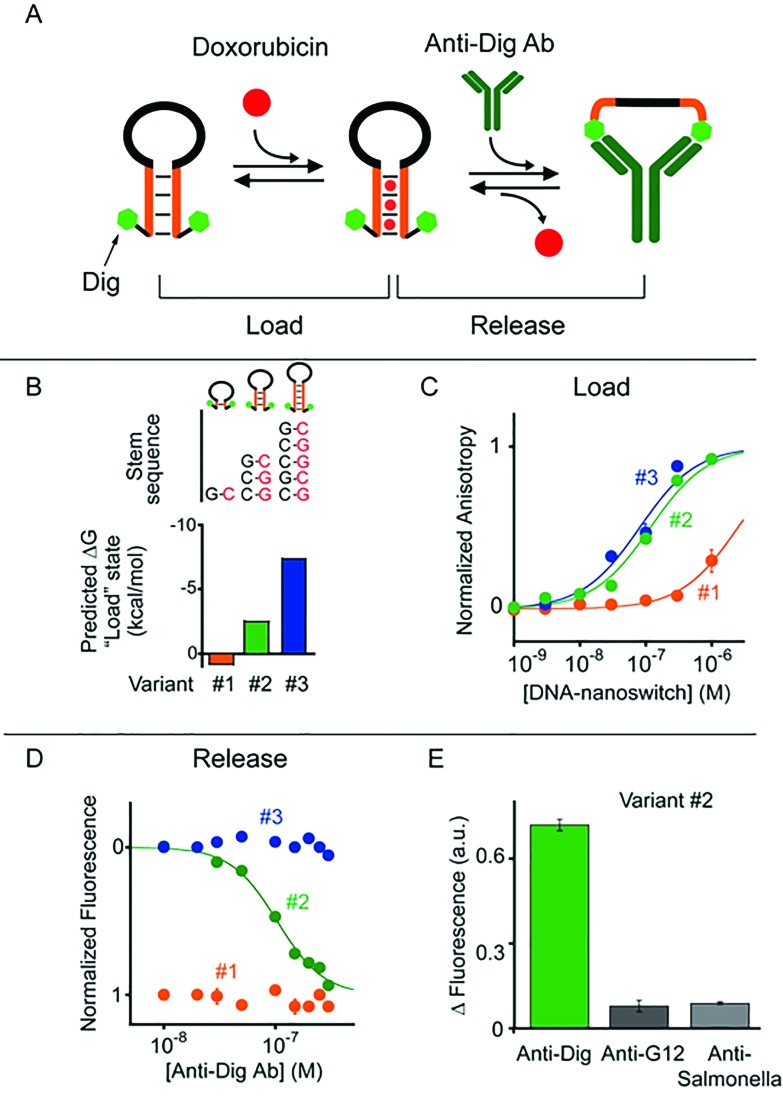
Antibody-regulated DNA-nanoswitches. (A) Doxorubicin is released from a “Load” state upon the binding of a specific antibody (here the anti-Dig antibody) to two recognition elements (here Dig) appended at the two ends of a DNA-nanoswitch. (B and C) We designed three variants of DNA-nanoswitches with different stabilities of the “Load” states (indicated are the free energies predicted using mfold) showing different doxorubicin intercalation efficiencies. (D) Doxorubicin release at increasing concentrations of anti-Dig antibody followed by steady-state fluorescence experiments. (E) Using variant #2, doxorubicin release occurs only in the presence of the specific antibody and no significant release is observed in the presence of other non-specific antibodies (at 300 nM). Here doxorubicin loading experiments have been performed by adding the relevant antibody-regulated DNA-nanoswitch to a solution of 100 nM of doxorubicin and measuring the fluorescence anisotropy signal of doxorubicin. For releasing experiments, increasing concentrations of anti-Dig antibody were added to an equimolar solution of doxorubicin and the DNA-nanoswitch (100 nM) and the doxorubicin signal was measured *via* steady-state fluorescence. In both cases the excitation and emission wavelengths were fixed at 480 nm (±7 nm) and 592 (±10) nm, respectively. All experiments were conducted at pH 7.0 in 50 mM sodium phosphate, 150 mM NaCl and 10 mM MgCl_2_ in a 100 μL cuvette at 25 °C. For further information about the experimental set up see the Experimental section.

To demonstrate the generality of our approach we designed a second class of allosterically controlled DNA-nanoswitches. In this case we designed a DNA-nanoswitch that can release intercalated doxorubicin in the presence of a specific antibody. We did so by designing a stem-loop doxorubicin-intercalating DNA-nanoswitch, terminally modified at both the 5′ and 3′ ends with the small molecule digoxigenin (Dig), which can be controlled by specific anti-Dig antibodies (Fig. 3). Binding of an anti-Dig antibody to the two Dig molecules at the two ends of the stem-loop, in fact, causes a conformational change that opens this secondary structure thus releasing the intercalated doxorubicin.^24^ Also, in this case we designed three different variants of this DNA-nanoswitch differing in their stem stability (Fig. 3A and B) and thus in their ability to be allosterically controlled by antibody binding. We found that the resultant doxorubicin loading profiles are consistent with the predicted energy gap between the “Load” (stem-loop) and “Release” (open) states (Fig. 3B). More specifically, while variants #2 and #3, which have GC-rich stems, show a similar doxorubicin loading efficiency, variant #1, which is designed to have a quite unstable “Load” state (Δ*G*_load_state_ = +0.8 kcal mol^–1^), shows a much poorer affinity towards doxorubicin (Fig. 3C).

To follow the release of doxorubicin upon anti-Dig antibody addition we used in this case steady-state fluorescence experiments (where the doxorubicin signal increases upon release) instead of anisotropy measurements because the latter were affected by the presence in the solution of the anti-Dig antibody. As expected, with variant #1, which showed a poor doxorubicin intercalation efficiency, we did not observe any change in the fluorescence doxorubicin signal upon the addition of increasing concentrations of anti-Dig antibody (Fig. 3D). In contrast, the variant (*i.e.* #3) that was designed to have a highly stable “Load” state (five GC pairs in the stem, Δ*G* = –7.4 kcal mol^–1^, Fig. 3B), despite exhibiting a good doxorubicin intercalation efficiency (Fig. 3C), prevents conformational opening and thus doxorubicin release in the presence of the anti-Dig antibody. As a result, we did not observe any significant doxorubicin release from this variant even at saturating concentrations of the anti-Dig antibody (Fig. 3D, blue). A variant showing intermediate stability of the “Load” state (*i.e.* variant #2) exhibits both efficient doxorubicin intercalation (Fig. 3C) and robust drug release upon anti-Dig antibody addition. More specifically, the presence of the anti-Dig antibody induces doxorubicin release with an observed EC_50_ of 100 ± 10 nM (Fig. 3D) and the variant is insensitive to other, non-specific antibodies (at 300 nM) (Fig. 3E). Also in this case, we can estimate the total maximum amount of doxorubicin released from the DNA-nanoswitch upon anti-Dig antibody binding (*i.e.* 4.3 pmol in a 100 μL volume) and we observe a similar load/release efficiency over a wide temperature range (from 25 to 40 °C) using variant #2 (Fig. S5 and S6†).

As a control experiment we first tested one variant (*i.e.* control #1) of such antibody-regulated DNA-nanoswitches that was terminally modified with only one Dig moiety at the 5′ end. Because the presence of just one Dig on the DNA-nanoswitch sequence prevents the antibody-triggered conformational opening, we did not observe any significant doxorubicin release from this control variant even at saturating concentrations of the anti-Dig antibody (300 nM, Fig. S7†). We finally performed a second control experiment on variant #2 by using, instead of the bivalent parent antibody, an anti-Dig monovalent Fab fragment that also recognizes Dig. The fact that no significant doxorubicin release was observed (Fig. S8†) confirms the proposed antibody-triggered doxorubicin release mechanism of our DNA-nanoswitches.

## Conclusions

In Nature, regulation of the activity of biological and cellular pathways is usually achieved through allostery. Allosteric propagation results in communication between distal sites in the protein structure and takes place through dynamic shifts of conformations thus affecting the equilibrium of macromolecular interactions. It also encodes specific effects on cellular pathways, and in this way it governs cellular response. Through this mechanism, tailored functions and biological activities triggered by biomolecular inputs are finely regulated in a concentration-dependent fashion. Because of this, synthetic biologists and bioengineers are intensely interested in recreating allostery in artificial systems. Allosteric nucleic acid catalysts, for example, have already been demonstrated to be useful tools not only for biosensing purposes but also as controllable therapeutic agents for gene therapy strategies or as molecular tools for controlling gene expression.^25^

Here we expanded on this theme by demonstrating a general strategy to achieve drug release that is allosterically regulated by a biological input. Our strategy is based on the design of conformational DNA-nanoswitches that flip from a “Load” state to a second active “Release” state in the presence of a specific biological input. We have applied this strategy to two DNA-nanoswitches that have previously been characterized for biosensing applications, and controlled them by transcription factors (here TBP) and antibodies, which represent a broader class of important markers for cancerous,^26^ neurodegenerative^27^ and autoimmune diseases.^28^ We note that the same approach could, in principle, be adapted to any input-induced DNA conformational switch that has been demonstrated to date, including those controlled by pH,^29^ aptamers' targets,^17b,30^ light,^31^ heavy metals,^17c,32^ and electronic inputs.^33^ While here we have focused our efforts on doxorubicin release, we note that the ability of DNA to bind to different therapeutic drugs (*i.e.* daunorubicin, actinomycin D, miRNA *etc.*) potentially opens the door to future application of this strategy for the controlled release of other DNA-binding molecular drugs.^34^

Finally, because several DNA-based vehicles and nanostructures have been associated with innovative approaches to enhance their transfection properties and overcome some of the current barriers in drug delivery,^35^ our strategy might allow extension of the functionality of these devices so that they can be regulated by different biological inputs (such as antibodies and proteins) and it could thus open the door for the next generation of DNA-based drug delivery systems.

## Experimental section

### Reagents

Antibodies were purchased from Roche Diagnostic Corporation, Germany (sheep polyclonal anti-Dig and sheep polyclonal anti-Dig Fab fragment), Biomedal, Spain (mouse monoclonal anti-G12), and Acris, Germany (mouse polyclonal anti-*Salmonella*). TATA-binding protein (TBP) was obtained by expression of recombinant, His-tagged proteins in *Escherichia coli*, as described previously.^36^ All of the proteins were aliquoted and stored at 4 °C for immediate use or at –20 °C for long-term storage. Reagents (NaCl, MgCl_2_, NaH_2_PO_4_, doxorubicin hydrochloride and Bovine Serum Albumin (BSA), all from Sigma-Aldrich, St Louis, Missouri) were used without further purification. HPLC-purified oligonucleotides were purchased from IBA, (Gottingen, Germany) or Biosearch Technologies (Risskov, Denmark).

The sequences of the DNA-nanoswitches regulated by the TATA-binding protein (TBP) are as follows:
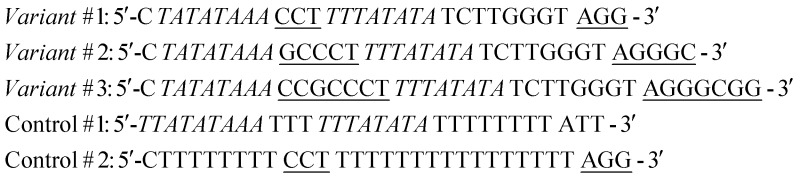


In the sequences above the underlined bases represent the stem portion of the “Load” state, while the italic bases represent the TBP recognition element and the stem of the “Release” state. Control #1 contains the TBP recognition element (italic) but lacks the stem portion which is responsible for doxorubicin intercalation. Control #2 contains the same doxorubicin-intercalating stem portion as variant #1 but it does not contain the TBP recognition element.

The sequences of the antibody-regulated DNA-nanoswitches were modified at the two extremities with digoxigenin (Dig) and have the following sequences:
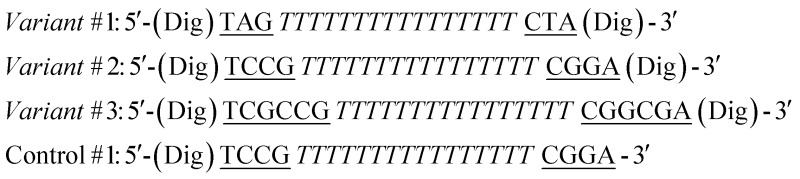


In the sequences above the underlined bases represent the stem portion, while the italic bases represent the loop. Control #1 is modified with only one copy of Dig.

### Fluorescence measurements

All fluorescence measurements were conducted at pH 7.0 in 50 mM sodium phosphate, 150 mM NaCl and 10 mM MgCl_2_ in a 100 μL cuvette at 25 °C unless otherwise noted. MgCl_2_ was used in these experiments to prevent non-specific electrostatic interactions between DNA and non-specific transcription factors or proteins.^37^ Loading curves were obtained at a fixed concentration of doxorubicin (100 nM) at increasing concentrations of the relevant DNA-nanoswitch. Release experiments were performed at an equimolar concentration of doxorubicin and the DNA-nanoswitch (100 nM), previously incubated at 25 °C for 20 min. In the case of TBP-triggered DNA-nanoswitches the release of doxorubicin was followed using fluorescence anisotropy measurements. For antibody-regulated DNA-nanoswitches doxorubicin release was followed using steady-state fluorescence measurements. Static fluorescence measurements were obtained using a Cary Varian Eclipse Fluorometer, with excitation at 480 (±5) nm and acquisition from 500 nm to 700 nm (±10). The intensity of the fluorescence emission at a fixed wavelength (*λ*_em_ = 592 nm) was measured. Fluorescence anisotropy experiments were obtained using a Fluoro4Max Horiba in L format-S side configuration and a true grating factor *G* = 0.59. The excitation and acquisition wavelengths were fixed at 480 nm (±7 nm) and 592 (±10) nm, respectively. The curves were fitted with the following simplified Langmuir equation:
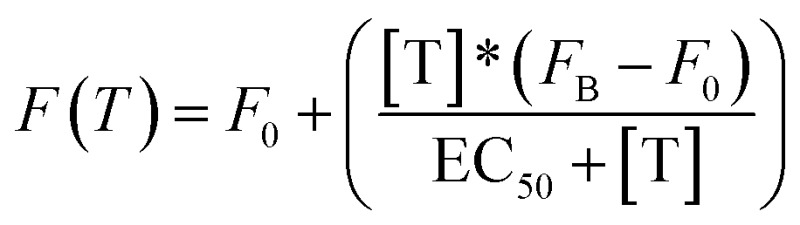
where *F*(*T*) = steady state/anisotropy fluorescence in the presence of different concentrations of target; *F*_0_ = background fluorescence; [T] = target concentration; *F*_B_ = fluorescence in the presence of a saturating concentration of target; EC_50_ = observed concentration of doxorubicin that gives a half-maximal response in the release experiments. For loading experiments the target is the DNA-nanoswitch; for releasing experiments the target is either TBP or the anti-Dig antibody.

### Melting curves analysis

Fluorescence melting curves were obtained in a 100 μL cuvette by using a Cary Varian Eclipse Fluorometer, with excitation at 488 (±5) nm and acquisition at 519 nm (±5) and using a solution containing a dual-labelled DNA-nanoswitch (100 nM) in 50 mM phosphate buffer, 150 mM NaCl and 10 mM MgCl_2_. The experiments were performed by heating from 20 °C to 90 °C at a rate of 1 °C s^–1^. The reported melting curves have been normalized through the use of the interpolation model.^38^ Melting temperatures (*T*_M_) have been obtained using the same model from the intersection of the calculated median and the experimental melting curve.

The following sequences (dual-labelled with AlexaFluor 488 and Black Hole Quencher) were used for the melting curve experiments:

Control #3 (analog to variant #1 of TBP-regulated nanoswitch): 5′-CTATATAAA (Alexa488) CCT TTTATATA TCTTGGGT AGG (BHQ1)-3′

Control #2 (analog to variant #2 of antibody-regulated nanoswitch): 5′-(Alexa488) TCCG TTTTTTTTTTTTTTTT CGGA (BHQ1)-3′
