# Supplementary material for: Allosteric DNA nanoswitches for controlled release of a molecular cargo triggered by biological inputs
Source: Chem Sci. 2016 Nov 3;8(2):914–20. doi: 10.1039/c6sc03404g (PMC5452262; doi:10.1039/c6sc03404g)
Supplement: Supplementary file 1 [file SC-008-C6SC03404G-s001.pdf]

Electronic Supplementary Material (ESI) for Chemical Science.
This journal is © The Royal Society of Chemistry 2016

Allosteric DNA nanoswitches for controlled release of a molecular cargo triggered by biological inputs

Marianna Rossetti[1], Simona Ranallo[1], Andrea Idili[1], Alessandro Porchetta[1] and Francesco Ricci*[1]*

[1] Chemistry Department, University of Rome, Tor Vergata, Rome, Italy.

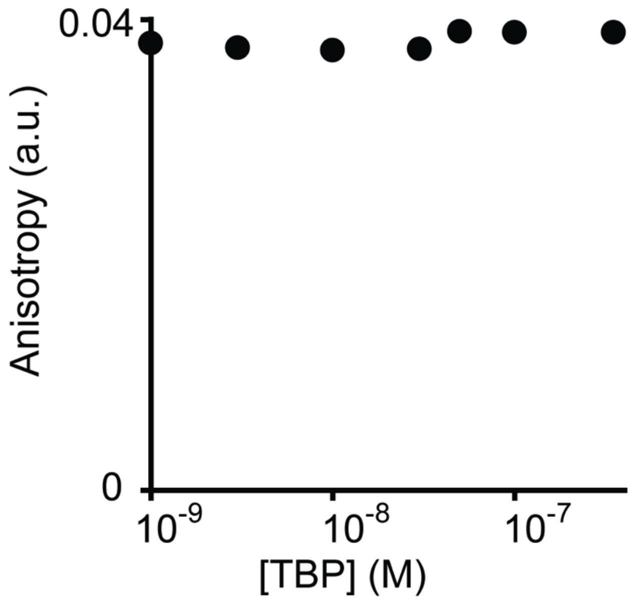

Figure S1. Interaction between Doxorubicin and TBP. Increasing concentrations of TBP have been added to a solution of doxorubicin (100 nM). The absence of significant anisotropy signal change of free doxorubicin (100 nM) confirms no specific interaction between free doxorubicin and TBP. The experiments were conducted at pH 7.0 in 50 mM sodium phosphate, 150 mM NaCl, 10 mM $MgCl_2$ in a 100 µL cuvette at 25°C. The excitation wavelength was fixed at 480 nm (± 7 nm) and emission at 592 (± 10) nm.

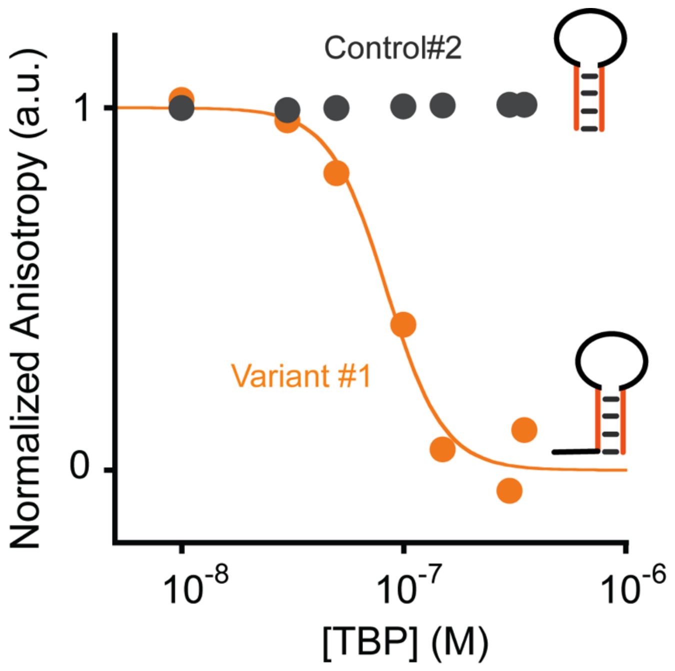

Figure S2. *Release* experiment performed on Control #2 of TBP-regulated DNA-nanoswitch. Control #2 has the same GC-rich stem portion of variant #1 but lacks the TBP recognition element in the sequence and shows no doxorubicin release upon TBP addition. See figure 2 and the methods section for experimental details.

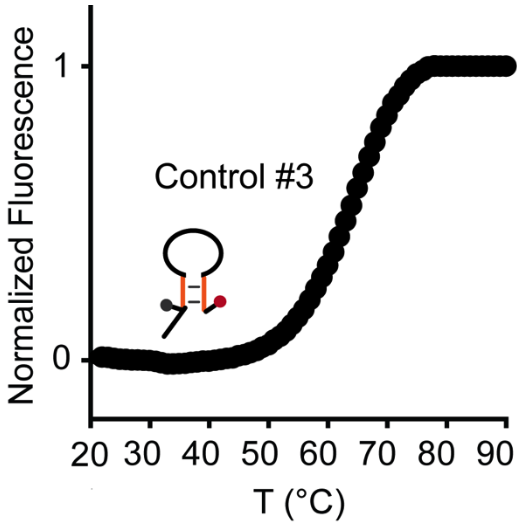

Figure S3. Melting curve of control #3, the dual-labelled analog of variant #1 of TBP-regulated DNA-nanoswitch, suggests that variant #1 is folded in the "Load" state between 20°C and 40°C (T_M = 63.4 °C).

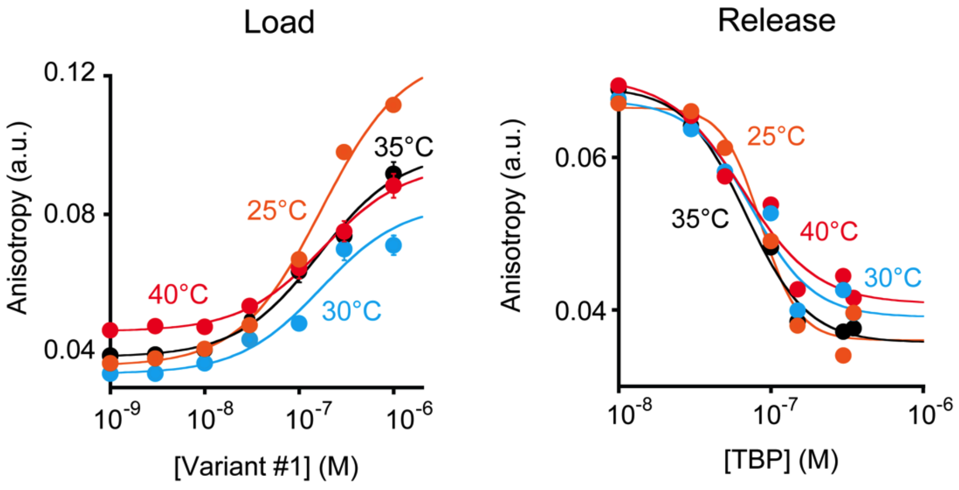

Figure S4. *Load/release* experiments performed with TBP-regulated DNA-nanoswitch (variant #1) at different temperatures. See figure 2 and the methods section for experimental details.

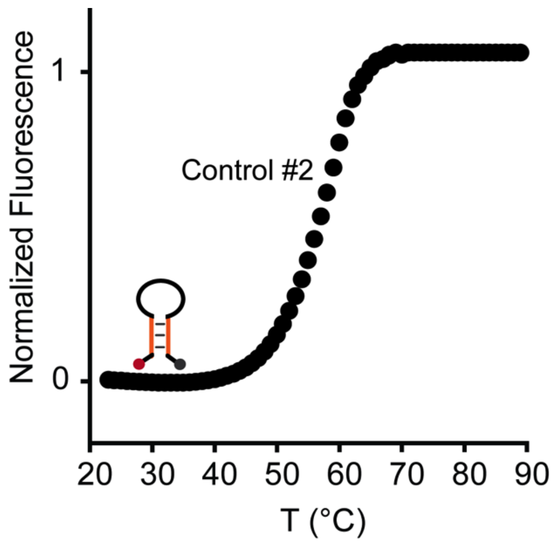

Figure S5. Melting curve of control #2, the dual-labelled analog of variant #2 of antibody-regulated DNA-nanoswitch, suggests that variant #2 is folded in the "Load" state between 20°C and 40°C (T_M= 56.2°C).

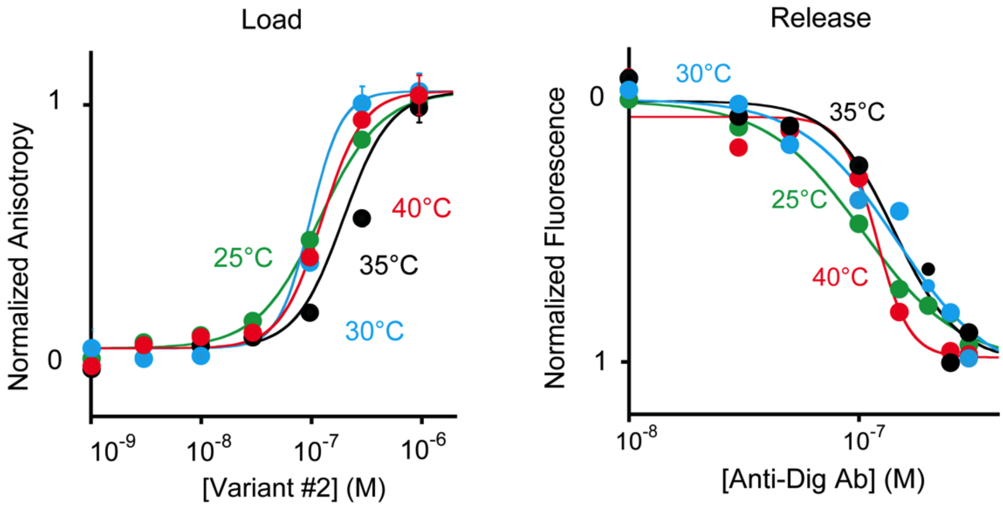

Figure S6. *Load/release* experiments performed with antibody-regulated DNA-nanoswitch (variant #2) at different temperatures. See figure 3 and the methods section for experimental details.

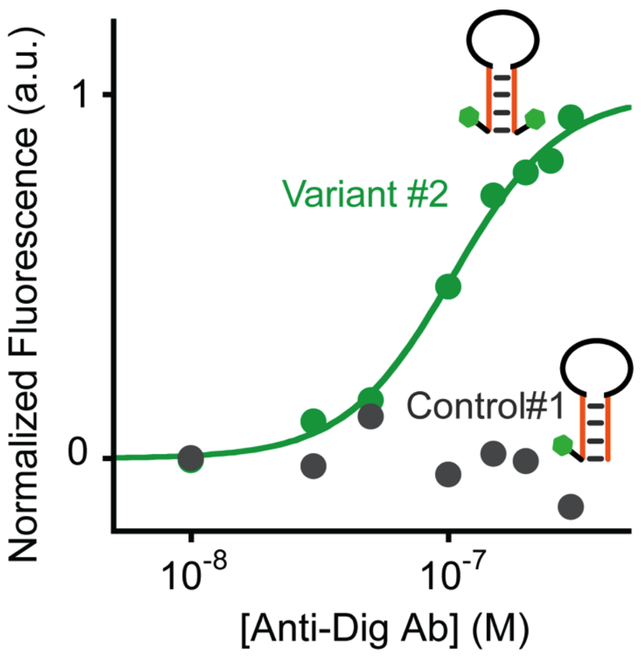

Figure S7. *Release* experiment performed on Control #1 of antibody-regulated DNA-nanoswitch. Control #1 has the same GC-rich stem portion of variant #2 but contains only one copy of Dig and shows no doxorubicin release upon anti-Dig antibody addition. See figure 3 and the methods section for experimental details.

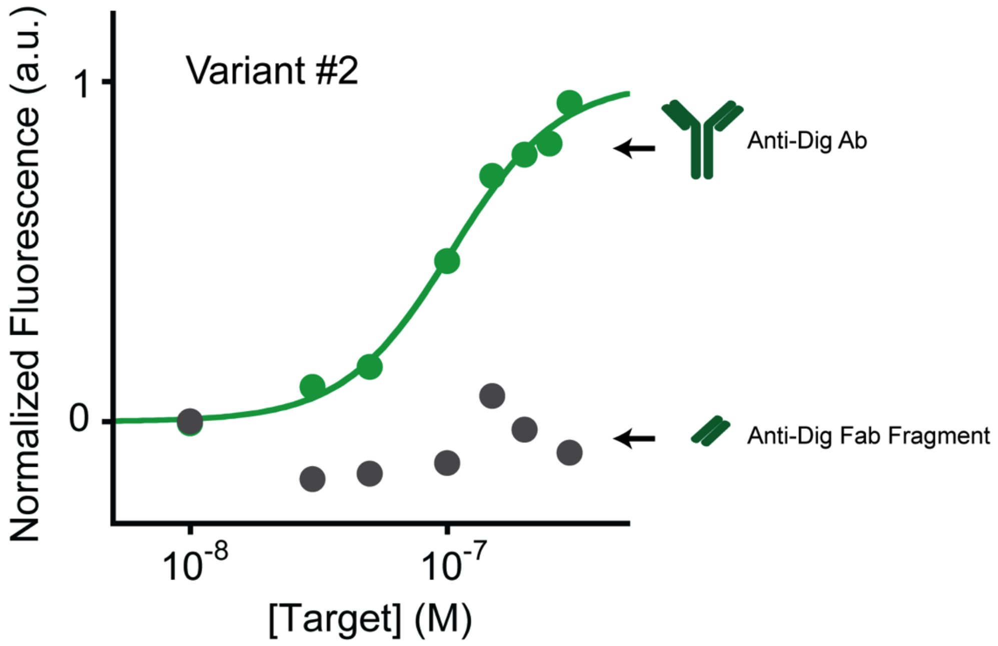

Figure S8. Control *release* experiment performed on antibody-regulated DNA-nanoswitch variant#2. The addition of increasing concentrations of anti-Dig Fab fragment does not result in any significant doxorubicin release. See figure 3 and the methods section for experimental details.
